# Effects of Vitreomacular Adhesion on Age-Related Macular Degeneration

**DOI:** 10.1155/2015/865083

**Published:** 2015-09-03

**Authors:** Eui Chun Kang, Hyoung Jun Koh

**Affiliations:** Department of Ophthalmology, Institute of Vision Research, Yonsei University College of Medicine, Seoul 120-752, Republic of Korea

## Abstract

Herein, we review the association between vitreomacular adhesion (VMA) and neovascular age-related macular degeneration (AMD). Meta-analyses have shown that eyes with neovascular AMD are twice as likely to have VMA as normal eyes. VMA in neovascular AMD may induce inflammation, macular traction, decrease in oxygenation, sequestering of vascular endothelial growth factor (VEGF), and other cytokines or may directly stimulate VEGF production. VMA may also interfere with the treatment effects of anti-VEGF therapy, which is the standard treatment for neovascular AMD, and releasing VMA can improve the treatment response to anti-VEGF treatment in neovascular AMD. We also reviewed currently available methods of relieving VMA.

## 1. Introduction

Age-related macular degeneration (AMD) is one of the leading causes of severe visual loss in developed countries and affects approximately 8.7% of elderly people >55 years old [1, 2]. Visual loss from this disease is mainly due to neovascular AMD or geographic atrophy (GA), and neovascular AMD accounts for 10% to 15% of patients with AMD [3]. Neovascular AMD is characterized by choroidal neovascularization (CNV), which is promoted by vascular endothelial growth factor (VEGF) and VEGF-A in particular [4]. A number of studies have identified risk factors for progression to neovascular AMD, including aging, cigarette smoking, and genetic factors [5]. Several studies have also postulated that vitreomacular adhesion (VMA) is associated with AMD pathogenesis or progression, and herein we review effects of VMA on neovascular AMD.

Krebs et al. first reported an increased incidence of VMA in eyes with neovascular AMD (18/50 [36%]) compared with dry AMD (4/57 [7%]) and controls (6/56 [11%]) [6]. Mojana et al. demonstrated a higher incidence of VMA in eyes with neovascular AMD compared with controls (27.8% versus 16%) [7]. In addition, a paired eye study revealed an increased rate of VMA in eyes with neovascular AMD compared with contralateral eyes which had dry AMD or no sign of AMD [8]. Roller et al. showed that progression of AMD, including geographic atrophy and CNV, was observed more frequently in nonvitrectomized eyes compared with vitrectomized eyes [9]. Together, these studies led us to speculate VMA might be a risk factor for the development of neovascular AMD and, if so, VMA might be a modifiable risk factor for neovascular AMD.

Currently, intravitreal anti-VEGF injections have been established as the standard treatment for neovascular AMD [10]. However, the study of ranibizumab in patients with subfoveal choroidal neovascularization secondary to age-related macular degeneration (SUSTAIN) showed that 26% of AMD patients were nonresponders to ranibizumab and showed no initial gain of vision or gain of vision during the course of treatment [11]. Another study reported that up to 45% of patients with neovascular AMD were nonresponders to bevacizumab [12]. Lee and Koh reported that VMA has an adverse effect on visual outcomes following anti-VEGF treatment, and studies by Üney et al. and Nomura et al. also showed the same results [13–15]. Thus, VMA might be a possible characteristic of nonresponders, and releasing VMA might be a feasible method to improve responses to anti-VEGF treatment in some eyes with VMA and neovascular AMD.

The purpose of this review is to understand the associations between VMA and neovascular AMD and suggest possible treatment options for VMA with neovascular AMD.

## 2. Definitions

### 2.1. Posterior Vitreous Detachment

The vitreous consists of water (98%) and structural macromolecules, including collagen and hyaluronan [16, 17]. The posterior vitreous is attached to the internal limiting membrane (ILM) of the retina by the macromolecular attachment complex including fibronectin and laminin. Posterior vitreous detachment (PVD) is defined as the dehiscence between the posterior vitreous and ILM. The vitreous gradually liquefies with age so that more than half of vitreous is liquid by the eighth decade [18]. PVD usually initiates as a focal detachment in the perifoveal macula, with persistent attachment to the fovea and optic nerve head [19]. Complete PVD describes the complete separation of the vitreous from the macula and optic nerve head. Incomplete or partial PVD naturally progresses to complete PVD.

Anomalous PVD may occur when vitreous liquefaction outpaces vitreoretinal dehiscence or abnormal adhesion is present between posterior vitreous and ILM. If anomalous PVD occurs in the macular area, macular hole or macular pucker may develop [16]. Retinal tears or retinal detachment can also occur if the abnormal adhesion exists in the peripheral retina in anomalous PVD.

### 2.2. Vitreomacular Adhesion

With the development of optical coherence tomography (OCT), the vitreomacular interface (VMI) can be conveniently assessed. The international vitreomacular traction study (IVTS) group proposed the classification of vitreomacular adhesion, traction, and macular hole according to anatomic features detected with OCT [20]. The vitreous and ILM are completely adherent at birth, so the concept of vitreoretinal adhesion is a normal anatomic state. However, the term VMA is clinically defined as the vitreous being attached within a 3-mm radius of the fovea, with surrounding separation of the cortical vitreous above the neurosensory retina [20, 21]. In addition, the retina should have no changes in surface contour or morphologic features on OCT. VMA can be subclassified as focal (≤1500 *μ*m) or broad (>1500 *μ*m) based on the size of the adhesion. Moreover, VMA should be referred to as concurrent when VMA is associated with retinal diseases (such as AMD, diabetic macular edema, or retinal vein occlusion) and isolated without retinal diseases.

### 2.3. Vitreomacular Traction

IVTS postulated that vitreomacular traction (VMT) is characterized by macular attachment of the vitreous cortex within a 3-mm radius of the fovea with distortion of the foveal surface, intraretinal pseudocyst formation, and elevation of the fovea from the retinal pigment epithelium (RPE). In VMT, the retinal layer should display no full-thickness interruptions [20]. VMT may be subclassified into focal (≤1500 *μ*m) or broad (>1500 *μ*m) like VMA according to the width of macular attachment. Furthermore, VMT can be termed concurrent or isolated, like VMA as previously described. However, it can be challenging to differentiate between VMT and VMA in eyes with neovascular AMD which have irregular retinal surfaces owing to underlying CNV and intraretinal cysts. In this review, we will focus on VMA but not VMT and discuss the effects of VMA in AMD.

## 3. The Roles of Vitreomacular Adhesion in Neovascular Age-Related Macular Degeneration

### 3.1. Inflammation and Oxidative Stress

Many immune-related genes that can induce inflammation, including age-related maculopathy susceptibility 2 (ARMS 2), complement factor H (CHF), and interleukin-8, are risk factors for AMD. Therefore, we speculate that inflammation can contribute to AMD pathogenesis [22, 23]. Inflammation can trigger tissue injury, oxidative stress, extracellular matrix remodeling, angiogenesis, and fibrosis in injured tissue [24]. In addition, retinal circulation has high metabolic rates with respect to oxygen consumption and the mitochondrial oxidative pathway, including phototransduction, neurotransmitter utilization, and protein/organelle transport [25, 26]. Dysregulation between oxidative stress and repair processes can lead to damage at the level of RPE cells, which cause AMD [27, 28]. VMA can induce chronic, low-grade inflammation with mechanical forces that can aggravate AMD [6]. However, there is currently no definite evidence whether VMA can cause the development of neovascular AMD or whether VMA is a consequence of the inflammation in neovascular AMD.

### 3.2. Tractional Macular Detachment, Retinoschisis, and Macular Edema Formation

Intraretinal cysts and retinoschisis can be induced by VMA according to Newton's third law: for every action, there is always an equal and opposite reaction [29]. When anomalous PVD gives rise to tractional force at the retina, there is an equal force in the opposite direction. This causes the retinal tissue of the retina to be pulled apart and leads to tractional macular detachment and retinoschisis formation [30]. In addition, interstitial tissue pressure decreases when the retina is pulled apart, which results in an influx of the fluid from blood vessels according to Starling's law of hydrostatic pressure [21]. This fluid influx contributes to the macular edema that is clearly evident in neovascular AMD.

### 3.3. Decreased Vitreous Oxygenation

The retina has dual blood supply from both choroidal and retinal circulation. Choroidal circulation primarily supplies the outer retina, which includes the highly metabolic photoreceptors and RPE, while retinal circulation supplies the inner retina [31]. However, some studies have proposed that the retina might be partially oxygenated by the vitreous [32, 33]. The speed of diffusion is inversely related to the viscosity of the vitreous. Therefore, vitrectomized eyes that have a lower viscosity than intact eyes show increased vitreous oxygenation in both humans and animals [34–36]. Complete PVD induced by ocriplasmin also increases vitreous oxygen levels in the vitreous cavity [37]. Conversely, VMA, which is mostly located over the area of the CNV in neovascular AMD [8, 38], may disturb oxygenation from the vitreous to the retina.

### 3.4. Increased VEGF and Proangiogenic Cytokines in front of the Macula

Increased VEGF can cause CNV in AMD and the current gold standard treatment for AMD is anti-VEGF treatment with ranibizumab or bevacizumab [10, 39]. In neovascular AMD, the prevalence of retinal vascular abnormalities is increased [40]. VEGF and other proangiogenic cytokines can diffuse into the vitreous cavity from these abnormal retinal vessels [41]. Vitreous collagen fibrils are altered with aging, and VEGF and other cytokines can be retained by binding to altered collagen fibrils between ILM and posterior vitreous cortex in VMA above the CNV area [42]. This can cause an increase in the concentration of VEGF and other proangiogenic cytokines in front of the macula and they can aggravate neovascularization and inflammation.

### 3.5. Expression of VEGF by Mechanical Stretch

Many studies have shown that mechanical stretching of the retina can induce VEGF expression [43–46]. A previous study supposed that VMA can disrupt choroidal blood supply to the retina and lead to hypoxia and result in increased VEGF levels [43]. Mechanical stress can also be an important regulator of gene expression, protein synthesis, growth, and the differentiation of many cell types [44]. Recent in vitro studies show that mechanical stress on RPE cells can induce elevated levels of succinate, which results in increased VEGF expression [45].

## 4. VMA and Neovascular AMD

It is generally accepted that there is an association between VMA and neovascular AMD. A recent meta-analysis on the prevalence of VMA in AMD reported that VMA in eyes with neovascular AMD, dry AMD, and normal controls were 22.6%, 9.5%, and 7.7%, respectively. Thus, eyes with neovascular AMD are 2.15 times more likely to have VMA than normal controls [47]. However, there is a controversy regarding prognosis after anti-VEGF treatment in VMA with neovascular AMD.

Some studies have reported that VMA is associated with poor visual outcome after anti-VEGF treatment for neovascular AMD. Lee and Koh reported that visual acuity after intravitreal anti-VEGF treatment decreased from 0.87 logarithm of the Minimum Angle of Resolution (logMAR) to 0.98 logMAR in eyes with VMA (3.87 injections per year) and improved from 0.82 logMAR to 0.72 logMAR in eyes without VMA (3.58 injections per year) between baseline and the 12 months of follow-up [13]. Üney et al. showed that eyes with VMA had a tendency to lose 4.9 early treatment diabetic retinopathy study (ETDRS) letters in eyes with VMA (3.5 injections per year) and gain 9.2 ETDRS letters in eyes without VMA (4.0 injections per year) after 12 months of anti-VEGF treatment [14]. Nomura et at. also described that visual acuity was unchanged from 0.42 logMAR to 0.39 logMAR in eyes with VMA (5.1 injections per year) but increased from 0.41 logMAR at baseline to 0.29 logMAR in eyes without VMA (5.2 injections per year) within the 12-month follow-up period [15].

A randomized, double-masked, active-controlled, multicenter study comparing the efficacy and safety of ranibizumab administered as two dosing regimens in patients with subfoveal choroidal neovascularization secondary to age-related macular degeneration (EXCITE) compared the efficacy of monthly versus quarterly ranibizumab injections after a loading phase for eyes with neovascular AMD and also investigated the influence of VMA on the efficacy of ranibizumab [48]. The EXCITE study group divided patients into three groups: (1) PVD, patients with PVD; (2) RELEASE, patients with VMA at first but not at last follow-up; and (3) VMA, patients with persistent VMA. In a protocol of quarterly injections after a loading phase (six injections per year), mean changes in ETDRS letters were +4.7 for PVD, +3.2 for RELEASE, and −0.2 for VMA within 12 months. These data were similar to results reported by Lee and Koh, Üney et al., and Nomura et al. from the viewpoint that eyes with VMA have poorer responses to anti-VEGF treatment [13–15].

However, in the monthly injection protocol group (12 injections per year) of the EXCITE study, the mean gains in ETDRS letters were +4.9 for PVD, +12.7 for RELEASE, and +7.5 for VMA. With continuous anti-VEGF injections, RELEASE and VMA groups, which include patients with VMA at baseline, showed better visual acuity than the PVD group after 12 months of follow-up [48]. They speculated that visual outcomes of neovascular AMD with VMA differed according to the frequency of intravitreal ranibizumab injections, and eyes with VMA in neovascular AMD can achieve favorable vision outcomes through an aggressive and continuous injection protocol.

Waldstein et al. investigated the influence of VMA on the efficacy of pro re nata (PRN) anti-VEGF injections after a loading phase. They reported that changes in ETDRS letters from baseline to the 12-month follow-up visit were +3.5 for PVD, +4.3 for RELEASE, and +6.3 for VMA, which were not significantly different [49]. In contrast to other studies that showed no significant differences in the number of anti-VEGF injections between eyes with VMA and without VMA, there were more injections in the RELEASE (6.6 injections per year) and VMA groups (5.3 injections per year) compared to the PVD group (4.9 injections per year).

Recently, the comparison of AMD treatments trials (CATT) study for 2 years of follow-up compared the visual acuity and number of injections according to the presence of VMA or VMT assessed by OCT [50]. In 598 eyes treated as needed protocol, there were 90 eyes (15.1%) with VMA at any time, 63 eyes (10.5%) with VMT at any time, and 445 eyes with neither VMA nor VMT at any time. Visual acuity outcomes between groups were not significantly different (*P* = 0.70). However, there were more frequent injections in VMA group and VMT group compared with the neither VMA nor VMT group (13.8 ± 0.73, 15.4 ± 0.87, and 12.9 ± 0.35, resp., *P* = 0.02).

Overall, it is probable that eyes with VMA and neovascular AMD may be less effective to loading plus PRN treatment, which is one of the most common protocols for neovascular AMD. A greater number of anti-VEGF injections in eyes with VMA and neovascular AMD might be helpful to achieve similar visual outcomes in eyes without VMA, and even favorable visual outcomes can be attained with continuous anti-VEGF injections. A strategy for reducing the number of anti-VEGF injections should also be considered in eyes with VMA and neovascular AMD, as continuous injections can cause an excessive financial burden and induce a greater chance of developing geographic atrophy which can lead to marked loss of visual acuity and function [51].

## 5. Treatment Options for Eyes with VMA and Neovascular AMD

### 5.1. Vitrectomy

Ikeda et al. first performed vitrectomy for 12 eyes of 11 patients with VMA and neovascular AMD. After 6 months, CNV regressed in six eyes (50%) and completely disappeared in two eyes (17%). Moreover, visual acuity improved in four eyes, was maintained in four eyes, and decreased in four eyes [52]. Mojana et al. performed vitrectomy in five eyes with VMT, and four of the five eyes showed an improvement in visual acuity and a decrease in central foveal thickness on OCT [7]. Furthermore, two case series reported that vitrectomy can induce CNV regression in patients with VMT and neovascular AMD [53, 54]. Sakamoto et al. showed that CNV can regress or disappear after vitrectomy (40/54 eyes) and CNV settled more significantly in eyes with PVD than in eyes without PVD [55]. Schramm et al. investigated the efficacy and safety of a core vitrectomy in patients with neovascular AMD treated with anti-VEGF therapy and they concluded that core vitrectomy might produce similar functional outcomes with respect to decreasing the number of intravitreal ranibizumab injections required over 48 weeks, even though it can induce more CNV bleeding [56].

All of these studies demonstrate that vitrectomy can improve functional and anatomical outcomes or reduce the number of anti-VEGF injections in eyes with VMA and neovascular AMD. Benefits of vitrectomy can be achieved by increasing oxygen diffusion from the anterior chamber to the vitreous cavity and diffusion of VEGF and other proangiogenic cytokines that are trapped between the posterior vitreous and ILM [7]. Furthermore, in vitrectomized eyes, the passage of anti-VEGF from the vitreous to subretinal space is not disturbed by the posterior vitreous cortex during anti-VEGF treatment. However, prospective studies are necessary to confirm the role of vitrectomy in AMD pathogenesis and progression.

### 5.2. Pharmacologic Vitreolysis

Vitreolytic agents are classified into (1) interfactants that can weaken VMA (e.g., dispase), (2) liquefactants that can induce vitreous liquefaction (e.g., hyaluronidase), or (3) a combination of both (e.g., plasmin, ocriplasmin, and tissue plasminogen activator) [57]. Of these agents, plasmin acts on glycoproteins, including fibronectin and laminin, without damaging the retina and has been widely studied. However, plasmin's use is limited in patients owing to its rapid autolytic properties and the fact that it requires activation by proenzymes and plasminogen, which are not commercially available.

Ocriplasmin (JETREA, ThromboGenics Inc., Iselin, NJ, USA) is an alternative to plasmin that was recently approved for the treatment of symptomatic VMA in the USA and European Union [58]. Ocriplasmin is a human serine protease that contains the catalytic domain of plasmin. Ocriplasmin is more stable than plasmin and can induce vitreous liquefaction and cleave the vitreoretinal interface by degrading “glue” proteins, including fibronectin and laminin [57–59].

The microplasmin for intravitreous injection-traction release without surgical treatment (MIVI-TRUST) study group showed that VMA was resolved in 26.5% of eyes treated by a single injection of ocriplasmin (125 *μ*g in 0.10 mL), compared with 10.1% of eyes in the sham injection group within 28 days [59]. VMA resolution can be achieved more often in younger patients (<65 years), patients with focal VMA (adhesion diameter ≤ 1500 *μ*m), phakic patients, and patients without epiretinal membranes. Moreover, visual acuity improvement was better in younger patients (<65 years) and patients with lower baseline visual acuity (Snellen equivalent < 20/50) [60].

Recently, a phase II clinical trial evaluating the safety and efficacy of ocriplasmin in eyes with VMA and neovascular AMD was performed [61]. Their data showed that VMA was released in 24.3% (18/74) of the eyes in the ocriplasmin injection group compared with 12.0% (3/25) of eyes in the sham injection group at day 28 after injection. In addition, the ocriplasmin injection group received fewer anti-VEGF injections compared with the sham injection group during the 12-month study period (4.4 injections versus 6.1 injections). However, their study showed no significant differences in VMA resolution or injection numbers between ocriplasmin and sham injection groups owing to the limited sample size.

It is hypothesized that ocriplasmin can release VMA and reduce the number of anti-VEGF injections required in neovascular AMD, but additional studies with larger sample sizes should be performed to confirm the efficacy of ocriplasmin in patients with VMA and neovascular AMD.

### 5.3. Gas Injection

Gross-Jendroska et al. found that perfluoropropane (C_3_F_8_) gas injection improved pigment epithelial detachment caused by AMD [62]. Kim et al. reported that all four patients achieved VMA release in neovascular AMD when intravitreal perfluoropropane gas was injected [63]. Moreover, there were no adverse events, such as endophthalmitis, cataract progression, increased intraocular pressure, intraocular hemorrhage, or retinal detachment, during follow-up. Rodrigues et al. reported that VMA was released in 40% of eyes (6/14) within 1 month and 60% of eyes (9/14) within 6 months by pure perfluoropropane injection [64]. Another case series also reported that pure perfluoropropane injection induced PVD without serious adverse effects [65]. Therefore, pure perfluoropropane injection can be an option for releasing VMA in neovascular AMD.

### 5.4. Intravitreal Injection

In the MIVI-TRUST study, sham injections with 0.10 mL saline were performed in the placebo group, and spontaneous resolution of VMA occurred in 10.1% of eyes 28 days after injection [59, 60]. This means that the sham injection itself can affect VMI via mechanical force, without the enzymatic activity of ocriplasmin. There are many studies that speculate that the intravitreal injection itself can alter intraocular structures, including the vitreous, retina, and anterior chamber, by mechanical force and other unknown mechanisms [66, 67].

In eyes with neovascular AMD, multiple anti-VEGF injections are necessary to maintain visual outcome. As a result of multiple intravitreal injections, more eyes achieve VMA resolution during the injection period. In the EXCITE study, about half of the eyes with VMA experienced release at 12 months (29/54 in the quarterly injection group, 19/31 in the monthly injection group) [47]. Despite the fact that this sample included some cases in which spontaneous resolution occurred in eyes with VMA during the treatment period, multiple injections can affect the release of VMA in eyes with neovascular AMD.

### 5.5. Combined Therapy (Anti-VEGF Plus Photodynamic Therapy)

One study compared the effects of anti-VEGF monotherapy and anti-VEGF plus photodynamic therapy (PDT) in eyes with VMA of neovascular AMD. The study concluded that compared to anti-VEGF monotherapy, anti-VEGF plus PDT helped reduce the number of injections in eyes with VMA. The anti-VEGF plus PDT group also showed a trend of superior visual outcome, but this was not clinically significant [49]. Even if PDT did not relieve the VMA in neovascular AMD directly, it can reduce the number of injections, thereby ameliorating the financial burden and serious adverse events.

## 6. Conclusion

Clinical data shows that the probability of having VMA is twice great in eyes with neovascular AMD than in normal eyes [47]. VMA can affect neovascular AMD by (1) inducing inflammation and oxidative stress, (2) formation of macular detachment, retinoschisis, and macular edema, (3) decreasing of vitreous oxygenation, (4) trapping VEGF and proangiogenic cytokines in front of the macula, and (5) mechanical stress induced VEGF production. As eyes with VMA and neovascular AMD have a tendency to generate a poorer response to routine PRN anti-VEGF injection protocols, more aggressive and continuous anti-VEGF injections might be required to achieve favorable visual outcomes in this cohort.

VMA release can improve responsiveness to anti-VEGF treatments, and the required number of anti-VEGF treatments decreases as a result. Release of VMA can be achieved by (1) vitrectomy, (2) ocriplasmin injection, (3) gas injection, or (4) the repeated injection procedure itself. To decrease the number of injections, combination therapy (PRN plus PDT) should be considered.
